# Minimal Rectal Toxicity in the Setting of Comorbid Crohn’s Disease Following Prostate Cancer Radiotherapy with a Hydrogel Rectal Spacer

**DOI:** 10.7759/cureus.1533

**Published:** 2017-08-01

**Authors:** Raj Singh, Philip S Jackson, Mollie Blake, James Cutlip, Sanjeev Sharma

**Affiliations:** 1 Department of Radiation Oncology, Joan C. Edwards School of Medicine, Marshall University; 2 Department of Radiation Oncology, St. Mary's Medical Center

**Keywords:** hydrogel spacer, prostate cancer, radiation therapy, inflammatory bowel disease, intensity-modulated radiotherapy (imrt), volumetric modulated arc therapy (vmat), radiation toxicity, crohns disease

## Abstract

We present one of the first cases of a prostate cancer (PCa) patient with inflammatory bowel disease (IBD) treated with intensity-modulated radiotherapy (IMRT) and a hydrogel rectal spacer. A 73-year-old male with a past medical history significant for Crohn’s disease (CD) and the recent diagnosis of T1cN0M0 high-risk PCa was referred for definitive radiotherapy. Given the patient’s history of CD and the possible increased risk of gastrointestinal (GI) toxicity and disease exacerbation, prior to IMRT, a hydrogel spacer was placed between the prostate and the anterior rectal wall to further minimize irradiation to the rectum. The patient then received IMRT (78 Gy/2 Gy fractions at a 100 percent isodose line). Over the course of treatment, Radiation Therapy Oncology Group (RTOG) Grade 1 GI toxicities of mild diarrhea were noted during the fifth and sixth weeks of treatment as well as an RTOG Grade 1 genitourinary (GU) toxicity of a decrease in the urinary stream that resolved with tamsulosin. At the 3, 6, 9, and 12-month follow-ups, bowel movements and urinary stream were reported to be at baseline with prostate-specific antigen (PSA) levels of 0.18 ng/mL and 0.03 ng/mL at the three and nine-month follow-ups, respectively. As such, this case report suggests that IBD patients with localized PCa may be viable candidates for radiotherapy given the promising results of hydrogel spacers in combination with IMRT in limiting rectal toxicity.

## Introduction

External beam radiotherapy (RT) is commonly used in the treatment of clinically localized prostate cancer (PCa). The disadvantages of RT include possible genitourinary (GU) and gastrointestinal (GI) symptoms [1]. This is especially relevant for PCa patients with inflammatory bowel disease (IBD), as IBD is considered to be a relative contraindication of RT because of the possible increased risk of GI toxicity and subsequent disease exacerbation [2]. With the advent of intensity-modulated radiation therapy (IMRT), less irradiation to the surrounding tissue and acceptable GI toxicity rates can be achieved as compared to conventional RT techniques for IBD patients with PCa [3]. Additionally, the recent introduction of hydrogel rectal spacers in clinical practice provides a novel approach for further minimizing radiation to the rectum for PCa patients at a higher risk of late toxicities. We present one of the first cases of a PCa patient with IBD treated with IMRT and a hydrogel rectal spacer.

## Case presentation

On routine examination, a 73-year-old male was found to have a prostate-specific antigen (PSA) level of 4.03 ng/mL while on finasteride. The elevated PSA prompted a biopsy that revealed 3 of 12 positive biopsy cores on the left side with Gleason scores of 6 (3+3), 7 (3+4), and 9 (4+5). Staging studies consisting of computerized tomography (CT) scans of the abdomen and pelvis and a bone scan were negative for evidence of nodal or distant metastasis. He was concurrently staged as T1cN0M0 high-risk prostate adenocarcinoma due to the Gleason 9 component.

The patient was started on leuprolide and referred for RT. His past medical history was significant for Crohn’s disease (CD) diagnosed eight years prior with colonoscopy and biopsy confirmation. The patient was generally asymptomatic with intermittent mild diarrhea, no prior history of blood or mucus in the stool, and no medical management for CD.

**Figure 1:**
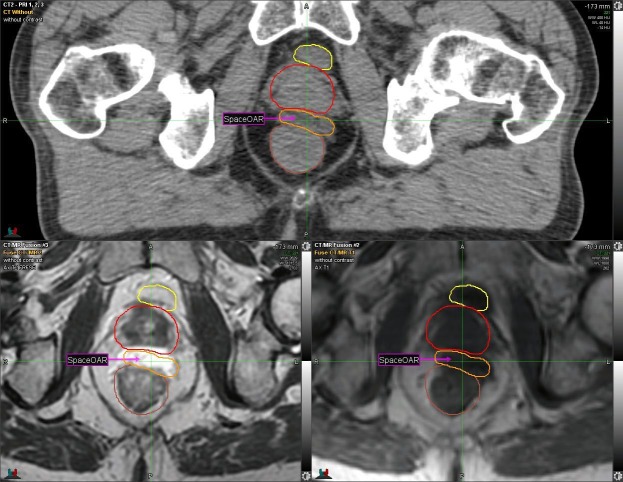
CT and MRI: Axial View Yellow = Bladder Red = Prostate Orange and Arrow = Spacer Brown = Rectum Computerized tomography: CT Magnetic resonance imaging: MRI

**Figure 2:**
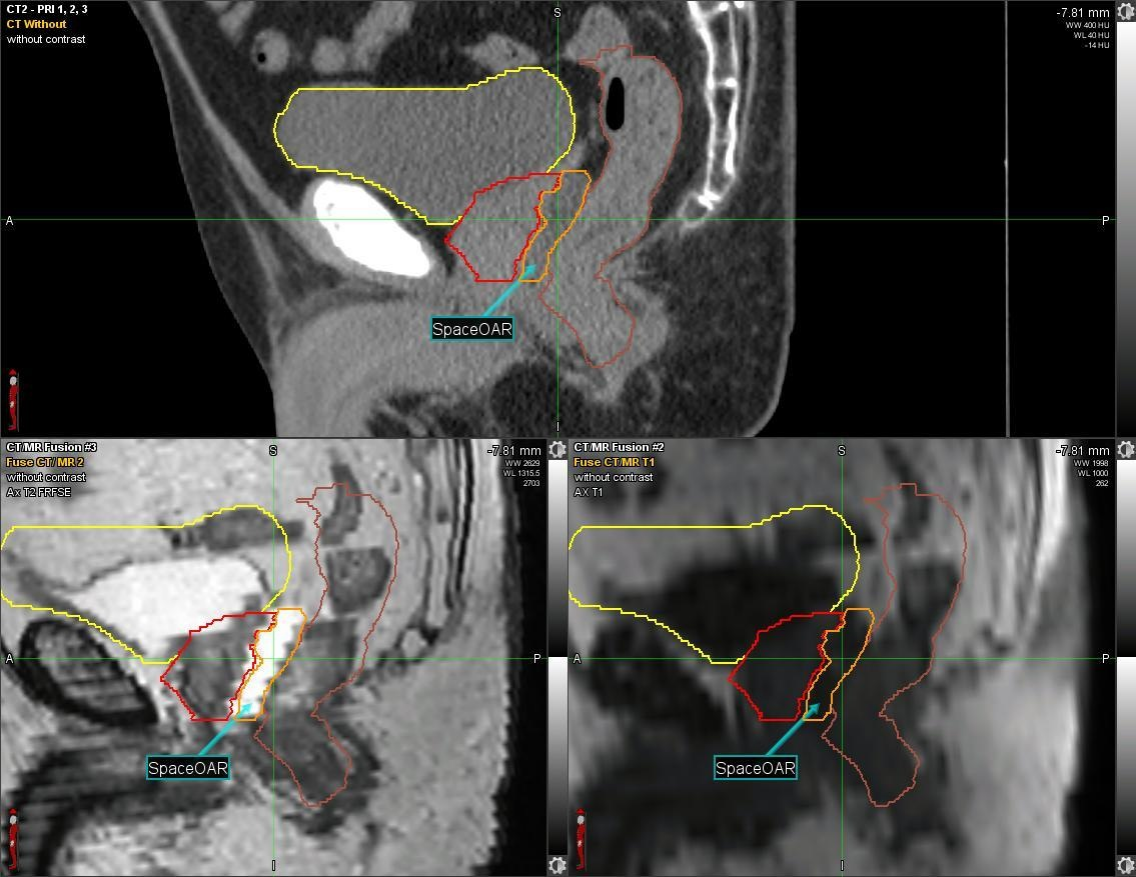
CT and MRI: Sagittal View Yellow = Bladder Red = Prostate Orange and Arrow = Spacer Brown = Rectum Computerized tomography: CT Magnetic resonance imaging: MRI

Given the patient’s history of CD, one week prior to RT, the SpaceOAR System (Augmenix; Waltham, MA) was utilized. SpaceOAR is a polyethylene glycol hydrogel that is injected into the perirectal fat and solidifies into an absorbable spacer that separates the prostate and the anterior rectal wall. Prior to spacer insertion, fiducials were placed. The hydrogel was then injected via transrectal ultrasound (TRUS) guidance with no perioperative complications and yielded a roughly 1.14 - 1.21 cm space between the anterior rectal wall and the prostate (Figure 1, Figure 2). 

Radiation Therapy

**Figure 3:**
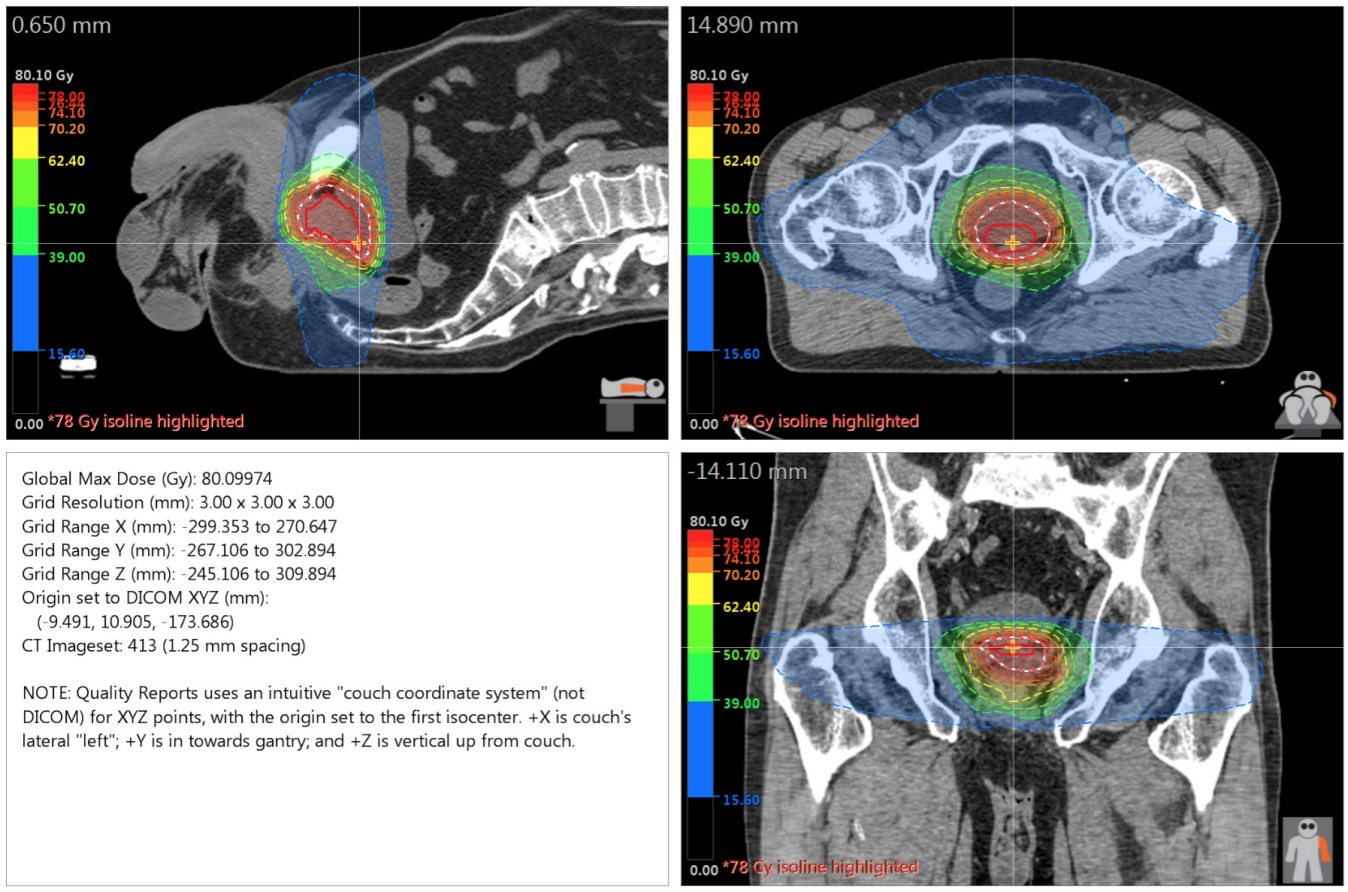
Dose Distribution for VMAT Plan Volumetric modulated arc radiotherapy: VMAT

The patient was treated in the supine position with RT delivered to both the prostate and seminal vesicles via volumetric modulated arc radiotherapy (VMAT) (78 Gy/2 Gy fractions at a 100 percent isodose line with two full arcs and 10 MV photons) with an Elekta Infinity linear accelerator (LINAC) (Elekta AB; Stockholm, Sweden). A Pinnacle treatment planning system (Phillips Radiation Oncology Systems; Fitchburg, WI) was utilized. Figure 3 outlines the dose distribution of the RT plan.

**Figure 4:**
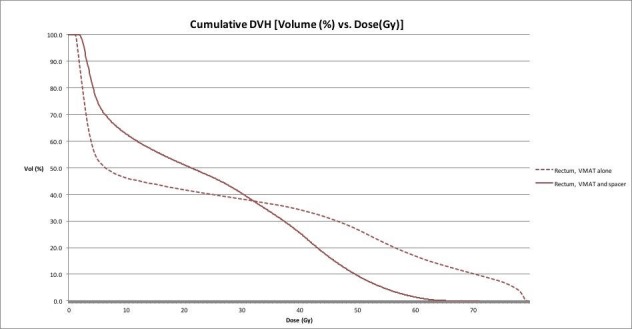
DVH Comparing VMAT with a Rectal Spacer and VMAT Alone Solid line = VMAT and rectal spacer Dashed line = VMAT alone Dose volume histogram: DVH Volumetric modulated arc radiotherapy: VMAT

A dose volume histogram (DVH) that compares rectal irradiation for a VMAT plan with a rectal spacer as against without a rectal spacer can be seen in Figure 4. A diagnostic CT scan was utilized to simulate the VMAT plan without a rectal spacer for the same patient.

Notably, the maximum dose (Dmax) received by the rectum was 60.4 Gy with spacer placement as compared to 78.7 Gy without the hydrogel spacer, with dosimetric benefits most evident at higher doses. A more detailed comparison of toxicity to the rectum can be found in Table 1. Of note, given that the VMAT plan without the spacer was simulated using a diagnostic CT, no bowel prep was utilized and this may be responsible for the differing isodose lines and, subsequently, the relatively higher doses received by larger rectal volumes with the rectal spacer.

**Table 1:** Comparison of Rectal Toxicity with VMAT Alone to VMAT with a Rectal Spacer Volumetric modulated arc radiotherapy: VMAT

Dose received by [x%] of rectum	VMAT + spacer	VMAT
1%	60.4 Gy	78.7 Gy
2%	58.5 Gy	78.5 Gy
5%	54.4 Gy	77.1 Gy
10%	49.6 Gy	70.4 Gy
15%	45.90 Gy	62.5 Gy
20%	43.0 Gy	56.5 Gy
25%	40.37 Gy	51.8 Gy
30%	37.2 Gy	46.7 Gy
35%	33.66 Gy	38.9 Gy
40%	30.0 Gy	25 Gy
45%	25.72 Gy	13.7 Gy
50%	20.34 Gy	6.6 Gy

Toxicities

Over the course of treatment, RTOG Grade 1 GI toxicities of mild diarrhea with no associated pain or cramping were noted during the fifth and sixth weeks. RTOG Grade 1 GU toxicities of mild decreases in urinary stream were also reported during the third week of treatment, which resolved with tamsulosin at subsequent follow-ups.

One week following the completion of treatment, the patient reported an RTOG Grade 1 GI toxicity of mild diarrhea. At the 3, 6, 9, and 12-month follow-ups, bowel movements were reported as being back to baseline, and the patient's urinary stream was intact following discontinuation of tamsulosin with no other GU symptoms reported. PSA levels of 0.18 ng/mL and 0.03 ng/mL were noted at the three-month and nine-month follow-ups, respectively.

## Discussion

We have presented a case that demonstrates that minimal rectal toxicity can be achieved in certain IBD patients with PCa. Murphy, et al. found medication use for IBD management as the single significant prognostic factor associated with the increased likelihood of acute ≥ Grade 2 GI toxicities in 21 PCa patients with IBD (57.1 percent vs. 7.7 percent; p = 0.03) with no increased five-year risk of late ≥ Grade 2 GI toxicities among IBD patients as compared to controls (HR = 1.19, 95 percent CI: 0.28 – 5.01) [4]. Notably, the patient presented in this case was not on pharmacologic therapy and, as such, had a favorable prognosis with regards to toxicity stemming from RT. Similarly, Gestaut et al. reported no incidences of > Grade 1 diarrhea or proctitis following IMRT without any post-radiation strictures at an average follow-up of 12 years [3].

In addition, rectal spacers provide a marked dosimetric advantage for limiting rectal toxicity. Multiple studies have documented significant declines in the amount of radiation delivered to the rectum for > 90 percent of patients receiving spacers and consequent reductions in the probability of GI toxicities (> 50 percent) [5-6]. Similarly, van Gysen et al. have previously reported an improvement in rectal dosimetry for PCa patients treated with VMAT (80 Gy in 40 fractions with Dmax = 85 Gy) with concurrent hydrogel spacer placement (V80 = 0.1 percent) as compared to controls (V80 = 7 percent; p < 0.001) [7].

This dosimetric benefit has promising clinical implications. A trial of 222 PCa patients treated with IMRT (79 Gy in 1.8 Gy fractions) and randomized to hydrogel spacer placement demonstrated a significant decrease in episodes of rectal pain (p = 0.02) and late GI toxicities (7 percent vs. 2 percent; p = 0.04). There was also a roughly 10 percent decline in the proportion of patients reporting poorer bowel-related quality of life in the spacer cohort at the 15-month follow-up [8]. A retrospective study by Pinkawa et al. of 167 PCa patients receiving RT (up to 76 Gy) found that patients receiving spacer placement were less likely to require treatment for bowel symptoms (0 percent vs. 11 percent; p < 0.01) or have a new issue with stool passage (0 percent vs. 12 percent; p < 0.01) [9]. Similarly, a randomized single-blind Phase III trial of 222 men treated with image-guided intensity modulated radiation therapy (IG-IMRT) (79.2 Gy/1.8 Gy fractions) noted that spacer placement resulted in significantly lower rates of reductions in bowel-related quality of life (40.9 percent vs. 14.0 percent; p = 0.002) at a median follow-up of roughly three years [10].

## Conclusions

IBD is considered to be a relative contraindication to definitive RT for clinically localized PCa. This report has demonstrated the successful use of IMRT with a hydrogel spacer for limiting radiation-induced bowel toxicity for an IBD patient with PCa. Following spacer placement and subsequent RT, no late GI or GU toxicities have been reported at the one-year follow-up. These findings, in combination with previous studies, suggest that IBD patients with PCa and favorable prognostic factors may be viable candidates for definitive RT given the promising results of spacers in combination with IMRT in limiting rectal toxicity.
